# Incidence and prognostic significance of newly-diagnosed atrial fibrillation among older U.S. veterans hospitalized with COVID-19

**DOI:** 10.1038/s41598-024-51177-6

**Published:** 2024-01-10

**Authors:** Darae Ko, Timothy M. Treu, Laura Tarko, Yuk-Lam Ho, Sarah R. Preis, Ludovic Trinquart, David R. Gagnon, Kevin M. Monahan, Robert H. Helm, Ariela R. Orkaby, Steven A. Lubitz, Nicholas A. Bosch, Allan J. Walkey, Kelly Cho, Peter W. F. Wilson, Emelia J. Benjamin

**Affiliations:** 1 Section of Cardiovascular Medicine, Boston Medical Center, Boston University Chobanian and Avedisian School of Medicine, Boston, MA USA; 2 Hinda and Arthur Marcus Institute for Aging Research, Hebrew SeniorLife, Harvard Medical School, 1200 Center Street, Boston, MA 02131 USA; 3 Massachusetts Veterans Epidemiology Research and Information Center (MAVERIC), VA Boston Healthcare System, Boston, MA USA; 4 Department of Biostatistics, Boston University School of Public Health, Boston, MA USA; 5 Tufts Clinical and Translational Science Institute, Tufts University, Boston, MA USA; 6 Institute for Clinical Research and Health Policy Studies, Tufts Medical Center, Boston, MA USA; 7 Division of Aging, Department of Medicine, Brigham & Women’s Hospital, Harvard Medical School, Boston, MA USA; 8 New England GRECC (Geriatric Research, Education, and Clinical Center), VA Boston Healthcare System, Boston, MA USA; 9 Cardiac Arrhythmia Service, Massachusetts General Hospital, Boston, MA USA; 10 Section of Pulmonary, Allergy, Sleep, and Critical Care Medicine, Department of Medicine, Boston University Chobanian & Avedisian School of Medicine, Boston, MA USA; 11 Atlanta VA Medical Center, Decatur, GA USA; 12 Division of Cardiology, Department of Medicine, Emory University School of Medicine, Atlanta, GA USA; 13 Department of Epidemiology, Boston University School of Public Health, Boston, MA USA

**Keywords:** Risk factors, Atrial fibrillation, Viral infection

## Abstract

Most prior studies on the prognostic significance of newly-diagnosed atrial fibrillation (AF) in COVID-19 did not differentiate newly-diagnosed AF from pre-existing AF. To determine the association between newly-diagnosed AF and in-hospital and 30-day mortality among regular users of Veterans Health Administration using data linked to Medicare. We identified Veterans aged ≥ 65 years who were hospitalized for ≥ 24 h with COVID-19 from 06/01/2020 to 1/31/2022 and had ≥ 2 primary care visits within 24 months prior to the index hospitalization. We performed multivariable logistic regression analyses to estimate adjusted risks, risk differences (RD), and odds ratios (OR) for the association between newly-diagnosed AF and the mortality outcomes adjusting for patient demographics, baseline comorbidities, and presence of acute organ dysfunction on admission. Of 23,299 patients in the study cohort, 5.3% had newly-diagnosed AF, and 29.2% had pre-existing AF. In newly-diagnosed AF adjusted in-hospital and 30-day mortality were 16.5% and 22.7%, respectively. Newly-diagnosed AF was associated with increased mortality compared to pre-existing AF (in-hospital: OR 2.02, 95% confidence interval [CI] 1.72–2.37; RD 7.58%, 95% CI 5.54–9.62) (30-day: OR 1.86; 95% CI 1.60–2.16; RD 9.04%, 95% CI 6.61–11.5) or no AF (in-hospital: OR 2.24, 95% CI 1.93–2.60; RD 8.40%, 95% CI 6.44–10.4) (30-day: 2.07, 95% CI 1.80–2.37; RD 10.2%, 95% CI 7.89–12.6). There was a smaller association between pre-existing AF and the mortality outcomes. Newly-diagnosed AF is an important prognostic marker for patients hospitalized with COVID-19. Whether prevention or treatment of AF improves clinical outcomes in these patients remains unknown.

Newly-diagnosed atrial fibrillation (AF) occurs in 5–10% of hospitalized patients with sepsis^1^. Among critically ill patients with sepsis admitted to intensive care units (ICU), newly-diagnosed AF was associated with 1.5–twofold increase in ICU length of stay^2,3^ and in-hospital mortality^1–3^. Newly-diagnosed AF also has been associated with poor prognosis in Coronavirus Disease 2019 (COVID-19). In two well-powered, multicenter studies of patients hospitalized with COVID-19 in New York City and its metropolitan areas from February to April 2020, incidence of newly-diagnosed AF was 4–11%, and newly-diagnosed AF was associated with 1.5- to 1.8-fold increased risk of in-hospital mortality^4,5^. In contrast, among patients hospitalized with COVID-19 from January 2020 to March 2021 in the American Heart Association COVID-19 Cardiovascular Registry, newly-diagnosed AF was not associated with in-hospital mortality after multivariable adjustment^6^. In these studies it is not clear how well pre-existing AF was differentiated from newly-diagnosed AF because the patients’ past medical history and prior diagnoses may not have been linked to the COVID-19 hospitalization.

Misclassification of pre-existing AF as new-onset AF may attenuate the strength of the association between newly-diagnosed AF and adverse outcomes in models adjusting for baseline cardiovascular comorbidities^7,8^. To accurately differentiate between pre-existing and newly-diagnosed AF, we analyzed national, longitudinal data from the United States (US) Veterans Health Administration (VHA) linked to Medicare data of patients hospitalized with COVID-19. Our primary objectives were to determine (1) incidence of newly-diagnosed AF in patients hospitalized with COVID-19; and (2) association between newly-diagnosed AF and in-hospital and 30-day mortality. Our secondary objective was to determine association between pre-existing AF and in-hospital and 30-day mortality.

## Methods

The datasets generated and/or analyzed during the current study are not publicly available because of VA policies regarding data privacy, but investigators with appropriate authorizations within the Department of Veterans Affairs can request data access. Data are however, available from the corresponding author upon reasonable request from investigators with appropriate authorizations. All procedures performed in studies involving human participants were in accordance with the ethical standards of the institutional and/or national research committee and with the 1964 Helsinki Declaration and its later amendments or comparable ethical standards. This study was approved, and the requirement for obtaining patient informed consent was waived, by the VHA Boston institutional review board.

We identified Veterans aged ≥ 65 years with a positive COVID-19 polymerase-chain-reaction (PCR) test for severe acute respiratory syndrome coronavirus 2 (SARS-CoV-2) from June 1, 2020 to January 31, 2022 using electronic health record and administrative claims data from the VHA Corporate Data Warehouse. To improve comorbidities and outpatient drug utilization capture, we linked VHA data to Medicare part A, B, and D data up to December 31, 2019. This work was approved by the institutional review board at VHA Boston and the requirement for patient informed consent was waived. We then applied the following eligibility criteria: (1) hospitalized for ≥ 24 h within ≤ 7 days before a positive PCR test for SARS-CoV-2 (*i.e.,* index hospitalization); (2) were regular users of VHA, defined as having ≥ 2 primary care visits within 24 months prior to the index hospitalization; and (3) no missing data on age, sex, and body mass index, which are minimum variables indicating regular care at VHA. We included regular users of the VHA services to improve the diagnostic specificity of newly-diagnosed AF vs. pre-existing AF.

We used International Classification of Diseases (ICD)-10 codes I48.xx to identify patients with AF. Patients without AF prior to the index hospitalization who were diagnosed with first AF during the hospitalization were classified as newly-diagnosed AF. Those with ≥ 1 inpatient or ≥ 2 outpatient diagnoses of AF within 24 months prior to the index hospitalization were classified as pre-existing AF. Patients without any diagnosis of AF either prior to or during the index hospitalization were classified as no AF.

Information on age, sex, self-reported race (American Indian or Alaskan Native, Asian, Black, Native Hawaiian or Other Pacific Islander, White) and ethnicity (Hispanic or not Hispanic), and body mass index was abstracted from inpatient or outpatient encounters most proximal to the index hospitalization. Race and ethnicity were included because prior studies have reported AF incidence and prevalence vary by race and ethnicity^9^.

Cardiovascular and non-cardiovascular comorbidities were defined using ICD-10 codes within 12 months prior to the index hospitalization. We defined comorbidities that may be associated with AF incidence and risk of in-hospital mortality: heart failure, hypertension, hyperlipidemia, diabetes, stroke or transient ischemic attack, coronary artery disease, peripheral vascular disease, dementia, chronic obstructive pulmonary disease, obstructive sleep apnea, and chronic kidney disease (Supplemental Table S1).

Exposure to oral anticoagulants was defined using prescription fills for all doses of warfarin, dabigatran, rivaroxaban, apixaban, and edoxaban within 180 days prior to the index hospitalization. Exposures to statins, digoxin, beta-blockers, calcium channel blockers, angiotensin converting enzyme inhibitors, angiotensin receptor blockers, AF-specific antiarrhythmics (i.e., amiodarone, dofetilide, dronedarone, flecainide, sotalol, and propafenone), and oral antiplatelets (i.e., aspirin, clopidogrel, prasugrel, ticagrelor) were defined using prescription fills within 365 days prior to the index hospitalization.

We defined presence of acute organ dysfunction on admission using a widely used claims-based organ dysfunction algorithm in severe sepsis^10^. The algorithm includes dysfunction in cardiovascular, respiratory, neurologic, hematologic, and renal systems (Supplemental Table S1). For medications specific to COVID-19, we included remdesivir, tocilizumab, and corticosteroids. Cardiovascular medications included rate-controlling agents, anti-arrhythmics, anti-hypertensives, and anticoagulants. Anticoagulants included intravenous heparin, low molecular weight heparin, subcutaneous heparin, apixaban, rivaroxaban, dabigatran, edoxaban, warfarin, and fondaparinux and were stratified into 3 mutually exclusive, hierarchical categories based on ≥ 1 dose received: treatment, prophylactic, and unknown dose.

Co-primary outcomes were all-cause in-hospital and 30-day mortality. Death information was ascertained from the National Death Index. Follow-up for 30-day mortality began on the first day of the index hospitalization.

Continuous variables were reported as mean ± standard deviation (SD) or median and [25th–75th percentile]. Categorical variables were reported as counts and percentages. We performed multivariable logistic regression analyses to generate marginal adjusted risks, risk differences (RD), and odds ratios (OR), adjusting for patient demographics and baseline comorbidities (i.e., age, sex, race, ethnicity, body mass index, heart failure, hypertension, hyperlipidemia, diabetes, stroke or transient ischemic attack, coronary artery disease, peripheral vascular disease, dementia, chronic obstructive pulmonary disease, obstructive sleep apnea, and chronic kidney disease) (model 1) and presence of acute organ dysfunction on admission (model 2). In secondary analyses, we examined association between pre-existing AF (vs no AF) and the outcomes.

All analyses were conducted using SAS, version 9.4; SAS Institute, Cary, North Carolina. Two-sided p-value < 0.05 was considered statistically significant. This study was approved by the VHA Boston Institutional Review Board, and waiver of informed consent was obtained.

## Results

**Figure 1:**
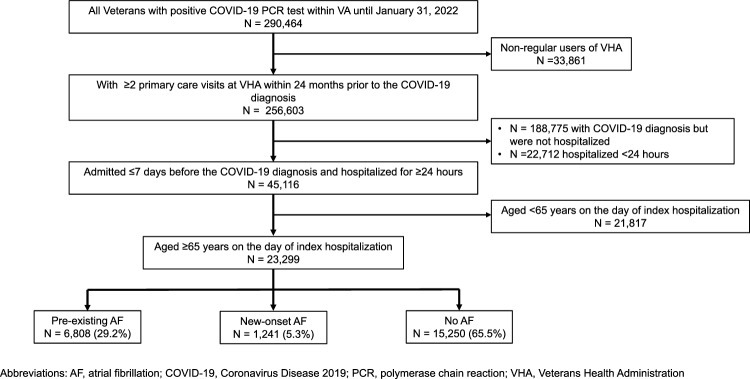
Flow Diagram for Cohort Selection. This diagram describes selection and exclusion of patients for our study cohort.

Our study cohort included 23,299 patients aged ≥ 65 years, who were hospitalized for ≥ 2 days with COVID-19 within VHA (Fig. 1). The mean age of the study cohort was 76 years (± 7.5 years) and 98% were men. In terms of racial/ethnic composition, 2.5% were American Indian or Alaskan Native, Asian, Native Hawaiian or Other Pacific Islander, 23% were Black, 6.8% were Hispanic or Latino, and 75% were White individuals. Among the patients without prior history of AF, 1,241 (7.5%) patients were newly diagnosed with AF during the index hospitalization. Pre-existing AF was present in 6,808 (29.2%) of the study cohort. Compared to patients with pre-existing AF, patients with newly-diagnosed AF were younger, more likely to be Black individuals, and less likely to have both cardiovascular and non-cardiovascular comorbidities. As expected, at baseline, the patients with pre-existing AF were more likely to be treated with beta-blockers, AF-specific antiarrhythmics, and oral anticoagulants (Table 1).

**Table 1:** Patient characteristics stratified by atrial fibrillation status.

	Newly-diagnosed AF (n = 1241)	Pre-existing AF (n = 6808)	No AF (n = 15,250)
Demographics			
Mean age (SD), years	76.8 (7.5)	77.9 (7.7)	75.1 (7.2)
≥ 75 years, n (%)	657 (52.9)	4082 (60.0)	6694 (43.9)
Women, n (%)	20 (1.6)	95 (1.4)	371 (2.4)
Race			
American Indian or Alaskan Native, Asian, Native Hawaiian or Other Pacific Islander, n (%)	38 (3.1)	163 (2.4)	373 (2.4)
Black, n (%)	243 (19.6)	1127 (16.6)	3993 (26.2)
White, n (%)	960 (77.4)	5518 (81.1)	10,884 (71.4)
Ethnicity			
Hispanic or Latino, n (%)	77 (6.2)	349 (5.1)	1148 (7.6)
Patient conditions			
Body mass index, kg/m^2^—mean (SD)	29.7 (6.5)	30.0 (7.1)	29.4 (6.4)
Chronic kidney disease, n (%)	494 (39.8)	3702 (54.4)	5908 (38.7)
Chronic obstructive pulmonary disease, n (%)	287 (23.1)	2549 (37.4)	3892 (25.5)
Coronary artery disease, n (%)	280 (22.6)	3304 (48.5)	3841 (25.2)
Dementia, n (%)	92 (7.4)	882 (13.0)	1658 (10.9)
Diabetes, n (%)	435 (35.1)	2628 (38.6)	5726 (37.5)
Heart failure, n (%)	131 (10.6)	2903 (42.6)	1922 (12.6)
Hyperlipidemia, n (%)	860 (69.3)	5574 (81.9)	11,247 (73.8)
Hypertension, n (%)	686 (55.3)	4689 (68.9)	9150 (60.0)
Obstructive sleep apnea, n (%)	210 (16.9)	2048 (30.1)	3061 (20.1)
Peripheral vascular disease, n (%)	64 (5.2)	799 (11.7)	1052 (6.9)
Stroke or transient ischemic attack, n (%)	120 (9.7)	1526 (22.4)	2167 (14.2)
CHA_2_DS_2_-VASc score—mean (SD)	3.0 (1.4)	4.1 (1.6)	3.1 (1.5)
Outpatient medications			
Angiotensin-converting enzyme inhibitor or Angiotensin receptor blocker, n (%)	556 (44.8)	3328 (48.9)	6910 (45.3)
*Antiarrhythmics, n (%)	4 (0.3)	853 (12.5)	58 (0.4)
Amiodarone, n (%)	3 (0.2)	559 (8.2)	44 (0.3)
Dofetilide, n (%)	0	85 (1.2)	0
Dronedarone, n (%)	0	23 (0.3)	2 (0.01)
Flecainide, n (%)	1 (0.1)	58 (0.9)	5 (0.03)
Propafenone, n (%)	0	14 (0.2)	1 (0.007)
Sotalol, n (%)	0	139 (2.0)	7 (0.05)
Beta-blocker, n (%)	503 (40.5)	4652 (68.3)	5994 (39.3)
Calcium channel blocker, n (%)	454 (36.6)	2342 (34.4)	5314 (34.8)
Digoxin, n (%)	2 (0.2)	306 (4.5)	19 (0.1)
Oral anticoagulants, n (%)	99 (8.0)	3887 (57.1)	818 (5.4)
Oral antiplatelets, n (%)	383 (30.9)	2382 (35.0)	5296 (34.7)
Statins, n (%)	767 (61.8)	4783 (70.3)	9741 (63.9)
^†^Acute organ dysfunction on admission	793 (63.9)	3649 (53.6)	7448 (48.8)
Cardiovascular, n (%)	320 (25.8)	1150 (16.9)	1947 (12.8)
Hematologic, n (%)	167 (13.5)	716 (10.5)	1365 (9.0)
Neurologic, n (%)	195 (15.7)	758 (11.1)	1577 (10.3)
Renal, n (%)	575 (46.3)	2349 (34.5)	5049 (33.1)
Respiratory failure, n (%)	91 (7.3)	337 (5.0)	647 (4.2)
In-hospital medications			
Angiotensin-converting enzyme inhibitor or Angiotensin receptor blockers n (%)	451 (36.3)	2479 (36.4)	5657 (37.1)
*Antiarrhythmics, n (%)	281 (22.6)	1149 (16.9)	276 (1.8)
Amiodarone, n (%)	279 (22.5)	906 (13.3)	258 (1.7)
Dofetilide, n (%)	0	73 (1.1)	1 (0.00)
Dronedarone, n (%)	1 (0.1)	20 (0.3)	3 (0.02)
Flecainide, n (%)	1 (0.1)	47 (0.7)	6 (0.04)
Propafenone, n (%)	0 (0.0)	14 (0.2)	2 (0.01)
Sotalol, n (%)	3 (0.2)	117 (1.7)	7 (0.05)
^‡^Anticoagulant	1201 (96.8)	6208 (91.2)	14,089 (92.4)
Treatment, n (%)	887 (71.5)	4539 (66.7)	3694 (24.2)
Prophylactic, n (%)	235 (18.9)	1319 (19.4)	8815 (57.8)
Unknown dose, n (%)	79 (6.4)	350 (5.1)	1580 (10.4)
Beta-blocker, n (%)	972 (78.3)	4991 (73.3)	6435 (42.2)
Calcium channel blocker, n (%)	593 (47.8)	2312 (34.0)	5329 (34.9)
Digoxin, n (%)	89 (7.2)	436 (6.4)	30 (0.2)
Corticosteroids, n (%)	918 (74.0)	4150 (61.0)	9663 (63.4)
Remdesivir, n (%)	677 (54.6)	2952 (43.4)	7223 (47.4)
Tocilizumab, n (%)	84 (6.8)	189 (2.8)	545 (3.6)
Length of stay, days – median (IQR)	10 (5, 18)	6 (4, 12)	6 (4, 11)
Admission to intensive care unit, n (%)	290 (23.4)	775 (11.4)	1642 (10.8)
Mechanical ventilation > 24 h after admission	283 (22.8)	546 (8.0)	1227 (8.0)

*CHA*_*2*_*DS*_*2*_*-VASc* a risk score for stroke in patients with AF which assigns 1 point for congestive heart failure, 1 point for hypertension, 2 points for age 75 year or older, 1 point for diabetes, 2 points for history of stroke or transient ischemic attack, 1 point for vascular disease including prior myocardial infarction or peripheral arterial disease, 1 point for age 65 to 74 years, and 1 point for female sex, *IQR* interquartile range, *SD* standard deviation.

*Some patients may have received ≥ 1 antiarrhythmic drug.

^†^Patients can have acute organ dysfunction in more than one system.

^‡^Anticoagulant medications were stratified into 3 mutually exclusive, hierarchical categories based on ≥ 1 dose received.

Acute organ dysfunction on admission was more common in patients with newly diagnosed AF. Overall, 47% of the patients in the study cohort received remdesivir, 3.5% received tocilizumab, and 63% received corticosteroids. Compared to the patients with pre-existing AF or no AF, the patients with newly-diagnosed AF were more likely to receive these COVID-specific medications. Any anticoagulant use was nearly universal. Compared to the patients with pre-existing AF and no AF, the patients with newly-diagnosed AF had longer hospital stay, were more likely to be admitted to ICU, and were more likely to be mechanically ventilated (Table 1).

Overall, 11% of the patients hospitalized with COVID-19 died prior to hospital discharge; 15% died within 30 days after the first day of the index hospitalization. In the multivariable model adjusting for age, sex, race, patient comorbidities, and presence of acute organ dysfunction on admission, the risks of in-hospital and 30-day death in newly-diagnosed AF were 16.5% and 22.7%, respectively (Table 2). For in-hospital death, newly-diagnosed AF was associated with adjusted RD of 7.6 and 8.4% and adjusted OR of 2.02- and 2.24 compared to pre-existing and no AF, respectively (Table 3). For 30-day death, newly-diagnosed AF was associated with adjusted RD of 9.0 and 10.2% and adjusted OR of 1.86 and 2.07 compared to pre-existing and no AF, respectively. Compared to no AF, pre-existing AF was associated with significant in-hospital and 30-day death adjusted RD of 0.8 and 1.2% and adjusted OR of 1.10 for each (Supplemental Table S2).

**Table 2:** Risks of death stratified by atrial fibrillation status.

		No. of events, n	Model 1* Adjusted risk (95% CI)	Model 2^†^ Adjusted risk (95% CI)
In-hospital death	Newly-diagnosed AF	288	22.3 (20.0–24.7)	16.5 (14.6–18.6)
	Pre-existing AF	852	11.0 (10.3–11.8)	8.9 (8.26–9.66)
	No AF	1496	9.9 (9.39–10.4)	8.1 (7.66–8.59)
30-day death	Newly-diagnosed AF	348	27.1 (24.7–29.7)	22.7 (20.4–25.0)
	Pre-existing AF	1202	15.1 (14.2–16.0)	13.6 (12.8–14.5)
	No AF	2061	13.7 (13.1–14.2)	12.4 (11.9–13.0)

*Model 1 is adjusted for patient demographics and heart failure, hypertension, hyperlipidemia, diabetes, stroke or transient ischemic attack, coronary artery disease, peripheral vascular disease, dementia, chronic obstructive pulmonary disease, obstructive sleep apnea, and chronic kidney disease.

^†^Model 2 is Model 1 adjusted for acute organ dysfunction on admission.

**Table 3:** Association between newly-diagnosed atrial fibrillation and mortality.

	Newly-diagnosed vs. Pre-existing AF				Newly-diagnosed vs. No AF			
	Model 1*		Model 2^†^		Model 1		Model 2	
	RD (95% CI)	OR (95% CI)	RD (95% CI)	OR (95% CI)	RD (95% CI)	OR (95% CI)	RD (95% CI)	OR (95% CI)
In-hospital death	11.2 (8.78–13.7)	2.31 (1.98–2.70)	7.58 (5.54–9.62)	2.02 (1.72–2.37)	12.4 (10.1–14.8)	2.62 (2.27–3.02)	8.40 (6.44–10.4)	2.24 (1.93–2.6)
30-day death	12.0 (9.35–14.7)	2.09 (1.81–2.42)	9.04 (6.61–11.5)	1.86 (1.60–2.16)	13.5 (10.9–16.0)	2.35 (2.06–2.69)	10.2 (7.89–12.6)	2.07 (1.80–2.37)

*AF* atrial fibrillation, *CI* confidence interval, *OR* odds ratio, *RD* risk difference.

*Model 1 is adjusted for patient demographics and heart failure, hypertension, hyperlipidemia, diabetes, stroke or transient ischemic attack, coronary artery disease, peripheral vascular disease, dementia, chronic obstructive pulmonary disease, obstructive sleep apnea, and chronic kidney disease.

^†^Model 2 is Model 1 adjusted for acute organ dysfunction on admission.

## Discussion

Our study has three main findings. First, in our national cohort of VHA patients hospitalized with COVID-19 up to January 31, 2022, the risk of newly-diagnosed AF was 5.3% and prevalence of pre-existing AF was 29.2%. Second, newly-diagnosed AF was associated with adjusted risks of 16.5% and 22.7% for in-hospital and 30-day mortality, respectively. Third, the patients with newly-diagnosed AF were younger and healthier than the patients with pre-existing AF. Newly-diagnosed AF was associated with approximately 10% increase in absolute risks and twofold increased odds of deaths compared to pre-existing AF. Compared to newly-diagnosed AF (vs. no AF), pre-existing AF (vs. no AF) was associated with much smaller increase in risks of death.

The most important strength of our study is that by studying regular users of VHA and linking VHA data to Medicare data, we were able to maximize the diagnostic specificity of newly-diagnosed AF vs. pre-existing AF. Our results clearly demonstrate that misclassification between newly-diagnosed AF from pre-existing AF would lead to a smaller association as compared to newly-diagnosed AF only. Indeed, potential misclassification of pre-existing AF as newly-diagnosed AF may explain why the study of the American Heart Association COVID-19 Cardiovascular Registry did not find a significant association between newly-diagnosed AF and in-hospital mortality after adjusting for demographics, baseline comorbidities, and severity of COVID-19 (hazard ratio [HR] 1.10, 95% CI 0.99–1.23)^6^. Misclassification of pre-existing AF as newly-diagnosed AF is possible when patients are admitted to hospitals where they have not received regular care or any care prior to the index hospitalization. In addition, the American Heart Association COVID-19 Cardiovascular Registry adjusted for severity of COVID-19 (e.g., new renal replacement therapy, ICU level care, use of vasopressors) even though these treatments may have been given *after* AF occurred, and thus are not confounders but rather mediators. It is important to adjust for severity of illness at baseline *prior* to AF diagnosis, which we attempted by adjusting for severity of illness at the time of admission.

Similar to sepsis, newly-diagnosed AF in COVID-19 likely carries prognostic implications beyond traditional organ dysfunction measures^11^. Accelerated endocardial fibrosis and electrical remodeling from infection and inflammation and physiologic and iatrogenic sympathetic overstimulation are the potential contributors for development of AF in sepsis^11^. Whether newly-diagnosed AF is simply a marker of poor outcomes or a direct contributor to poor outcomes is unknown. Compared to pre-existing AF, new-onset AF in sepsis may be more likely to present with difficult to control heart rate, refractory hypotension, pulmonary edema, myocardial ischemia, and heart failure^11,12^. For example, in a study of patients admitted of ICU, 25% of patients with newly-diagnosed AF and 13% of patients with pre-existing AF had heart rates greater than 150 beats per minute^12^. Hemodynamic instability was present in 37% of newly-diagnosed AF and 10% of pre-existing AF patients^12^. A prior clinical trial demonstrated safety and effectiveness of using a short-acting intravenous beta-blocker in reducing the risk of atrial and ventricular arrhythmias in sepsis^13^. Whether prevention or treatment of AF with the short-acting intravenous beta-blocker improves clinical outcomes in patients with sepsis has not been studied in clinical trials. In addition, the best rate and rhythm control management for newly-diagnosed AF in sepsis has not been studied in clinical trials. Our study as well as others’^1–3^ call for prospective randomized studies to generate definitive evidence on these questions.

The results of our study should be interpreted in the context of the study’s limitations. First, there may have been ascertainment bias in AF diagnosis. Newly-diagnosed AF is often paroxysmal, and the likelihood of its detection increases with the duration of cardiac rhythm monitoring. Given the need to minimize the risk of contact transmission of the virus and the need to limit utilization of healthcare resources early in the pandemic, it is possible that patients with severe disease were more likely to undergo telemetry monitoring and therefore were more likely to have detected AF. Second, we were not able to perform survival analysis adjusting for time-varying covariates because we were unable to determine the exact timing of AF onset. Third, we were unable to determine AF burden in pre-existing AF in this retrospective observational study, and the association between newly-diagnosed AF vs. pre-existing AF and clinical outcomes may differ for different levels of AF burden in pre-existing AF. Fourth, although echocardiographic features (*e.g.,* left atrial size, left ventricular wall thickness, left ventricular ejection fraction) may have been associated with both increased risk of AF and worse clinical outcomes, we did not have access to echocardiographic data. Fifth, patients with subclinical pre-existing AF may have been misclassified as newly-diagnosed AF. Sixth, our analyses were restricted to US predominantly White male VHA inpatients and results may not be generalizable to other countries, women, and adults younger than 65 years. Additionally, our study results may not be generalizable to Veterans who do not receive regular primary care visits within VHA. Finally, because of the observational study design, our study cannot establish causality between newly-diagnosed AF and prognosis. Strategies to prevent new-onset AF and its effect on outcomes in COVID-19 would require randomized controlled trials.

### Supplementary Information

Supplementary Tables.

## Data Availability

The datasets generated and/or analyzed during the current study are not publicly available because of VA policies regarding data privacy, but investigators with appropriate authorizations within the Department of Veterans Affairs can requests for data access. Data are however, available from the corresponding author upon reasonable request from investigators with appropriate authorizations.

## References

[1] Walkey AJ, Wiener RS, Ghobrial JM, Curtis LH, Benjamin EJ (2011). Incident stroke and mortality associated with new-onset atrial fibrillation in patients hospitalized with severe sepsis. JAMA.

[2] Klein Klouwenberg PM, Frencken JF, Kuipers S (2017). Incidence, predictors, and outcomes of new-onset atrial fibrillation in critically ill patients with sepsis. A cohort study. Am. J. Respir. Crit. Care Med..

[3] Moss TJ, Calland JF, Enfield KB (2017). New-onset atrial fibrillation in the critically ill. Crit. Care Med..

[4] Mountantonakis SE, Saleh M, Fishbein J (2021). Atrial fibrillation is an independent predictor for in-hospital mortality in patients admitted with SARS-CoV-2 infection. Heart Rhythm.

[5] Musikantow DR, Turagam MK, Sartori S (2021). Atrial fibrillation in patients hospitalized with COVID-19: Incidence, predictors, outcomes and comparison to influenza. JACC Clin Electrophysiol..

[6] Rosenblatt AG, Ayers CR, Rao A (2022). New-onset atrial fibrillation in patients hospitalized with COVID-19: Results from the American Heart Association COVID-19 cardiovascular registry. Circ. Arrhythm. Electrophysiol..

[7] Aydemir S, Aksakal E, Aydınyılmaz F (2022). Does new onset and pre-existing atrial fibrillation predict mortality in COVID-19 patients?. Egypt Heart J..

[8] Spinoni EG, Mennuni M, Rognoni A (2021). Contribution of atrial fibrillation to in-hospital mortality in patients with COVID-19. Circ. Arrhythm. Electrophysiol..

[9] Virani SS, Alonso A, Benjamin EJ (2020). Heart Disease and Stroke Statistics-2020 update: A report from the American Heart Association. Circulation.

[10] Angus DC, Linde-Zwirble WT, Lidicker J (2001). Epidemiology of severe sepsis in the United States: Analysis of incidence, outcome, and associated costs of care. Crit. Care Med..

[11] Bosch NA, Cimini J, Walkey AJ (2018). Atrial fibrillation in the ICU. Chest.

[12] Kanji S, Williamson DR, Yaghchi BM, Albert M, McIntyre L (2012). Epidemiology and management of atrial fibrillation in medical and noncardiac surgical adult intensive care unit patients. J. Crit. Care.

[13] Kakihana Y, Nishida O, Taniguchi T (2020). Efficacy and safety of landiolol, an ultra-short-acting β1-selective antagonist, for treatment of sepsis-related tachyarrhythmia (J-Land 3S): A multicentre, open-label, randomised controlled trial. Lancet Respir. Med..

