# Supplementary material for: Incidence and prognostic significance of newly-diagnosed atrial fibrillation among older U.S. veterans hospitalized with COVID-19
Source: Sci Rep. 2024 Jan 10;14:952. doi: 10.1038/s41598-024-51177-6 (PMC10781702; doi:10.1038/s41598-024-51177-6)
Supplement: Supplementary file 1 — Supplementary Tables. [file 41598_2024_51177_MOESM1_ESM.docx]

**SUPPLEMENTARY MATERIALS**

**Incidence and Prognostic Significance of Newly-Diagnosed Atrial Fibrillation Among Older U.S. Veterans Hospitalized with COVID-19**

Darae Ko, MD, MSc; Timothy M. Treu, MPH; Laura Tark o, MPH; Yuk-Lam Ho, MPH; Sarah R. Preis, MPH, ScD; Ludovic Trinquart, PhD; David R. Gagnon, MD, PhD; Kevin M. Monahan, MD; Robert H. Helm, MD, FHRS; Ariela R. Orkaby, MD, MPH; Steven A. Lubitz, MD, MPH; Nicholas A. Bosch, MD, MSc; Allan J. Walkey, MD, MSc; Kelly Cho, PhD; Peter W.F. Wilson, MD; Emelia J. Benjamin, MD, ScM

**TABLE OF CONTENTS**

| **Supplemental Table S1** | Page 1 | Variable definitions |
| --- | --- | --- |
| **Supplemental Table S2** | Page 2 | Association between pre-existing atrial fibrillation and in-hospital and 30-day mortality |

**Supplemental Table 1.** Variable Definitions

| **Conditions** | **ICD-10 codes** |
| --- | --- |
| Acute organ dysfunction | Cardiovascular: I95.1 - I95.9, R57, R65.21  Acute respiratory failure: G47.30, G47.31, G47.33, CPT 94660, E0470 - E0472, E0561, E0562, E0601  Neurologic: F05, F06.0 - F06.4, F06.8, F53.0 - F53.1, G93.1, G93.4, I67.83  Hematologic: D65., D68.8, D68.9, D69.51, D69.59, D69.6  Renal: N17 |
| Atrial fibrillation | 427.3, I48 |
| Chronic kidney disease | An outpatient eGFR < 60 in the past year with a second measure of <60 between 90 days and 2 years of the first. |
| Chronic obstructive pulmonary disease | 490 - 496, 510, 519.11, J40 - J45, J47, J66 - J67, J82, J84, J98.2, J98.3, J98.9 |
| Coronary artery disease | 410, 411, 413, 414, I20 - I25 |
| Dementia | 290, F01.5, F02.8, F03.9, F10.27, F10.97, F13.27, F13.97, F18.17, F18.27, F18.97, F19.17, F19.27, F19.97, G30, G31.09, G31.83 |
| Diabetes | 250, E08-E11, E13 |
| Heart failure | 428, I50 |
| Hyperlipidemia | 272.0 - 272.3, E78.0 - E78.5 |
| Hypertension | 401.0, 401.1, 401.9, I10 |
| Peripheral vascular disease | 443.9, I73.9 |
| Obstructive sleep apnea | 780.51, 780.53, 780.57, G47.3 |
| Stroke or transient ischemic attack | 362, 388.02, 431-438, 997.02, G45 - G46, H34.0 - H34.239, H93.099, I60-I63, I65-I69, I97.8, V12.54, Z86.73 |

**Supplemental Table 2.** Association between pre-existing atrial fibrillation and mortality

|  | **Pre-existing AF vs. No AF** | | | |
| --- | --- | --- | --- | --- |
|  | Model 1* | Model 1 | Model 2^†^ | Model 2 |
|  | **RD (95% CI)** | **OR (95% CI)** | **RD (95% CI)** | **OR (95% CI)** |
| In-hospital death | 1.16 (0.23, 2.09) | 1.12(1.02-1.24) | 0.82 (0.03, 1.62) | 1.10 (1.00-1.22) |
| 30-day death | 1.46 (0.39, 2.52) | 1.11(1.02-1.21) | 1.19 (0.19, 2.19) | 1.10 (1.01-1.20) |

Abbreviations: AF, atrial fibrillation; CI, confidence interval, OR; odds ratio; RD, risk difference

*Model 1 is adjusted for patient demographics and patient comorbidities.

^†^Model 2 is Model 1 adjusted for acute organ dysfunction on admission.
